# Cellulose nanofiber from sunflower heads and pomegranate peels as sustainable reinforcing agents in biopolymer-based films for active packaging of bread

**DOI:** 10.1038/s41598-026-57588-x

**Published:** 2026-06-18

**Authors:** Attia A. Yaseen, Ahmed M. Youssef, Ragab Abouzeid, Fathy M. Mehaya, Salah H. Salem, Heba M. Amer, Ayman A. Mohammad

**Affiliations:** 1 Food Technology Department, National Research Centre, Dokki, Cairo, 12622 Egypt; 2 Packaging Materials Department, National Research Centre, Dokki, Cairo, 12622 Egypt; 3 Center for Converging Sciences and Emerging Technology (CoSET), Benha National University (BNU), Al Obour, 13518 Egypt; 4 Cellulose and Paper Department, National Research Centre, Dokki, Cairo, 12622 Egypt; 5 Food Toxicology and Contaminants Department, National Research Centre, Dokki, Cairo, 12622 Egypt; 6 Medicinal and Aromatic Plants Research Department, National Research Centre, Dokki, Cairo, 12622 Egypt

**Keywords:** Pomegranate peels, Sunflower heads, Essential oils, Bioactive packaging, Cellulose nanofibers, Antimicrobial activity, Pan bread, Biochemistry, Biotechnology, Chemistry, Materials science, Nanoscience and technology

## Abstract

Pomegranate peels and sunflower heads were used to prepare cellulose nanofibers (CNFs) via chemical and physical processes. The CNFs were characterized using FT-IR, TEM, and XRD, then incorporated into carboxymethyl cellulose (CMC) with clove, cumin, or cinnamon essential oils to form bioactive edible coating films. TEM revealed homogeneous nanofibers (diameters 6–15 nm), while XRD showed crystallinity of 69% (sunflower) and 72.5% (pomegranate). Essential oils were evaluated for chemical properties and antimicrobial activities. GC-MS identified eugenol (85.65%) and cinnamaldehyde (84.51%) as major constituents of clove and cinnamon oils, respectively. Clove oil showed the highest total phenolics and antioxidant activity, whereas cinnamon oil exhibited the strongest antimicrobial activity. Mechanical optimization was performed using CMC films reinforced with sunflower-derived CNFs, the maximum increases in tensile strength (from 10.4 to 28.5 MPa) and Young’s modulus (from 450 to 1603 MPa) at 10% CNFs loading, representing increases of 174% and 256%, respectively. Bread coated with films containing the cinnamon/cumin oil mixture had the lowest microbial counts after 72 h of open-air storage (bacteria: 9.4–9.5 × 10^4^ CFU/g; mold/yeast: 7.0-7.5 × 10^3^ CFU/g), which were lower than those of the uncoated control. Bread coated with pomegranate CNFs and clove oil showed the slowest weight loss and best freshness retention during the tested 72 h storage period. Organoleptic evaluation gave coated bread higher scores for crust color and aroma than uncoated controls. The CNF/essential oil containing coatings helped maintain bread quality and lowered microbial counts relative to the uncoated control during the tested 72 h open-air storage. This study demonstrates that agricultural wastes can be valorized through CNF extraction and that CNF-reinforced bionanocomposites with essential oils are promising candidates for short-term bioactive food-coating applications.

## Introduction

Nowadays, agricultural waste and food processing by-products are the most global issues due to their negative impact on the food security, ecosystem and economy. Annually; about 1.3 billion tons of food byproducts are generated, alongside up to 50% of agricultural produce lost worldwide. These residues, when improperly disposed of or discarded, represent huge amounts of environmental pollutants and lead to serious unhygienic climate. However, such untapped residues represent valuable resources that can play an important role in triggering the creation of several value-added new products^1–3^. Agro-industrial byproducts have gained attention as sustainable resources for producing high-value materials^4,5^. Among various valorization strategies, the production of cellulose nanofibers (CNFs) from lignocellulosic wastes aligns with circular economy principles^6,7^.

In this context, cellulose nanofibers (CNFs) are eco-friendly valuable substances with exceptional mechanical properties and versatile applications^8^. They are derived from the cellulosic materials, which are predominant components of agro-wastes^9^. Sunflower heads and pomegranate peels are two abundant underutilized residues. Sunflower heads contain 35–45% cellulose, 15–20% pectin, 5–10% hemicellulose, and 3–5% lignin^10^, while pomegranate peels comprise 22–41% lignin, 16–22% cellulose, and 5–13% hemicellulose^11,12^. Both materials are currently considered environmental pollutants^13,14^, yet their high cellulose content makes them promising sources for CNF extraction. CNFs possess tunable surface chemistry, high strength, and good barrier properties, making them attractive for edible food coatings and packaging^15,16^. Latterly, edible coatings have been applied for extending the shelf life of highly perishable foods, however biopolymer-based packaging materials still encounter several challenges^17^. These biopolymers often lack the mechanical robustness and generally have poor water vapor and microbial resistant properties^18,19^.

Thus, optimizing biopolymer properties remains a key research focus to meet the industrial applications on the economic scale^20,21^.Hydrophobic and nanofiller reinforcement strategies have implemented to improve the coating performance of biopolymer-based films^17,22^. Cellulose nanofibers emerged as a promising reinforcing agent for tailoring the coating properties of biofilms^23^. Also, essential oils represent another strategy to improve aroma, antioxidant, antimicrobial and mechanical properties of biofilms to meet specific application requirements in active packaging methods^24^. The bakery sector, particularly bread, suffers high post-harvest losses (9.7–14.4%)^25,26^, necessitating effective shelf-life extension strategies. Edible coatings incorporating CNFs and essential oils offer a potential solution^23^.

In this context, previous studies have explored CNF extraction from various residues and their incorporation into biopolymer-based films, but most have focused on single biomass sources. For instance, Oun and Rhim^27^ reported that adding 5 wt% CNF to CMC films improved tensile strength by only 23% and elastic modulus by 27%. Similarly, Rincon et al.^28^ observed a 70% tensile strength increase in CMC/CNF films with gallic acid. Regarding essential oil-based antimicrobial coatings for bread, log reductions of 2.5–3.2 log (CFU) have been reported for clove and cinnamon oils, while active chitosan/BCNF/AgNPs films reduced mold counts from 7.91 log CFU/g in controls to below detection after 15 days^29,30^. However, to the best of our knowledge, limited studies have evaluated CNFs derived from sunflower heads and pomegranate peels as agro-waste-derived reinforcing/bioactive components in the same bread coating study. In the present work, antioxidant activity was measured for the essential oils, mechanical optimization was conducted for sunflower-CNF-reinforced CMC films, and the antimicrobial effectiveness of the final coating formulations was evaluated through bread-storage tests. This study aimed to valorize pomegranate peels and sunflower heads by extracting CNFs and utilizing them to prepare bioactive edible coating formulations combined with selected essential oils. The edible coating formulations were then applied to pan bread to assess short-term quality retention and microbial spoilage control during 72 h of open-air storage. The study also compared coating formulations containing pomegranate or sunflower derived CNFs in bread-quality tests, while the mechanical optimization of CMC/CNF films was performed using sunflower-derived CNFs.

## Materials and methods

### Material

Pomegranate fruits (*Punica granatum* L.) (cv. ‘Wonderful’) of uniform size and color were purchased from the neighborhood market (Cairo, Egypt) at mature stage. Fully developed capitula (flower heads) of sunflower. Giza-102 (*Helianthus annuus* L.) at the physiological maturity stage was obtained from special farm, Giza, Egypt. Taxonomic identification was confirmed by the supplier. Clove (*Syzygium aromaticum* (L.) flower buds, cinnamon (*Cinnamomum verum* J. Presl) bark, and cumin (*Cuminum cyminum* L.) seeds were procured from a certified local supplier (Cairo, Egypt). The plants were identified by Medicinal and Aromatic Plant Dept. at National Research Centre. A voucher sample (HASA 2023, HASV 2023, HACC 2023), respectively was deposited at the Herbarium of National Research Centre. Pomegranate peels were obtained by manual separation of arils, and the peels were washed with distilled water, then dried (60 °C, 48 h) and milled into a fine powder (particle size < 500 μm) using a laboratory grinder. Sunflower heads were cleaned from stalk and seed residues, and the receptacle tissues were manually separated, dried at 60 °C and ground into a fine powder (particle size < 500 μm) using a laboratory grinder. Herbal materials were cleaned and ground using a laboratory mill to pass through a 40-mesh sieve.

tested microorganisms used for studying antimicrobial activity included Gram-positive bacteria *(Staphylococcus aureus* ATCC 13565, Bacillus cereus EMCC 1080, and *Listeria monocytogenes* ATCC 19115) kindly provided by the Collection of the Dairy Microbiological Lab, NRC, Egypt. Gram-negative bacteria (*Salmonella typhi* ATCC 15566, *Escherichia coli* O157:H7 ATCC 51659, and *Pseudomonas aeruginosa* NRRL B-272), yeast (*Candida albicans* ATCC 10231), and fungal strains (*Aspergillus flavus* NRRL 3357, A. ochraceus ITAL 14, A. niger ATCC 16888, *Penicillium verrucosum* BFE 500, *Fusarium verticillioides* ITEM 10027, and F. *proliferatum* MPVP 328) were kindly provided by the Marine Toxins Lab, NRC, Egypt.

### Preparation and characterization of cellulose nanofibers

#### Isolation of cellulose from solid residues

Delignification of pomegranate peel and sunflower head powders was carried out using acidified sodium chlorite method as described by^31^. Briefly, 5 g of each sample was suspended in 80 mL distilled water containing 1.5 g sodium chlorite (NaClO_2_) and 1 mL acetic acid (CH_3_COOH) and heated at 80 °C for 1 h. This step was repeated twice, and the resulting pulps were filtered and washed thoroughly with distilled water until neutral pH was reached, and several batches were conducted to obtain sufficient cellulose pulp. The cellulose yield, calculated gravimetrically as the percentage of oven-dried isolated cellulose pulp relative to the initial dry biomass weight, was lower for pomegranate peel than for sunflower head, with values of 29.8% and 35%, respectively.

#### Preparation of TEMPO-oxidized cellulose nanofibers

Surface functionalized CNFs were synthesized using 2,2,6,6-tetramethyl-1-piperidinyloxy (TEMPO) oxidation method as described by^32^. The process involved suspending cellulose samples (10 g) in distilled water (750 mL) containing TEMPO (25 mg) and sodium bromide (0.25 g) under magnetic stirring at room temperature. Sodium hypochlorite solution was added at a dosage of approximately 12 mmol NaClO per gram of dry cellulose. The pH of the slurry was adjusted to 10.5 and maintained at this value throughout the reaction by dropwise addition of 0.5 M NaOH. Oxidation was allowed to proceed for 2 h, and the reaction endpoint was identified when no further decrease in pH was observed. The reaction was then quenched by adjusting the suspension to neutral pH (pH 7.0) with hydrochloric acid. The oxidized cellulose was thoroughly washed with deionized water by filtration. The washed suspension was subsequently homogenized at a pulp concentration of 2% using a high-shear homogenizer (T-T18 ULTRA-TURRAX) operated at 10,000 rpm to obtain cellulose nanofibers (CNFs). After TEMPO-mediated oxidation and homogenization, the CNF yield was about 91% based on the dry weight of the extracted cellulose.

#### Fourier Transform Infrared analysis (FTIR)

FTIR spectral analysis of CNF samples was performed using a Tensor 27 FTIR spectrometer (Bruker Optics, Wissembourg, France) equipped with a single-reflectance horizontal diamond ATR-Golden Gate system (Bruker Optics). Samples were pressed directly against the surface of the diamond crystal and scanned in the range from 4000 to 650 cm⁻¹ with 32 scans against an air background spectrum.

#### X-ray Diffraction (XRD)

The XRD analyses of CNFs were performed at room temperature using Philips X-ray diffractometer with monochromatic Cu Kα radiation (λ = 0.154 nm). Diffraction patterns were collected across a 2θ range of 5° to 60° using a step size of 0.04°. The interplanar spacing was calculated according to Bragg’s law. The crystallinity index was determined using the Segal method, based on the intensity of the (200) reflection at 2θ = 22° (I_200_) and the minimum intensity between the (200) and (110) reflections at 2θ = 18° (I_am_), as expressed in the following equation^33^.

$$\:\mathrm{C}\mathrm{r}\mathrm{y}\mathrm{s}\mathrm{t}\mathrm{a}\mathrm{l}\mathrm{l}\mathrm{i}\mathrm{n}\mathrm{i}\mathrm{t}\mathrm{y}\:{\%}=\left(1-\frac{{I}_{am}}{{I}_{200}}\right)\times\:100$$

In this method, I_200_ corresponds to the combined contribution of crystalline and amorphous regions, whereas I_am_ reflects the amorphous fraction of the material.

#### Transmission Electron Microscopy (TEM)

The morphological characteristics of CNFs were examined using a transmission electron microscope (JEOL JEM-2100, Tokyo, Japan) operated at 160 kV. A dilute CNF dispersion (0.002 wt%) was prepared, and an aliquot (25 µL) was deposited onto a copper specimen grid and allowed to stand for 10 min. The grid was stained with 3% phosphotungstic acid and dried for 30 min prior to observation. Fibril diameter and length were quantified from the TEM images using ImageJ software.

### Extraction and characterization of essential oils

#### 2.3.1 Hydro-distillation extraction technique

Three hundred grams of cumin seeds, cinnamon bark, and clove buds were individually subjected to hydro-distillation using Clevenger-type units with a sample-to-water ratio of 1:10 (w/v) for 3 h. The resulting essential oils were collected in sealed vials, flushed with nitrogen and stored at − 18 °C. The essential oil yields were 3.75% (w/w) for cumin, 1.20% (w/w) for cinnamon, and 7.5% (w/w) for clove.

#### Gas chromatography–mass (GC-MS) analysis of essential oils

GC-MS analysis was performed using an Agilent 7890B Gas Chromatograph coupled with an Agilent 5977 A Mass Spectrometer Detector and equipped with an HP-5MS capillary column (30 m × 0.25 mm i.d., 0.25 μm film thickness). An aliquot of 1 µL of each essential oil sample was injected with a split ratio of 1:10 at an injector temperature of 220 °C. Helium was used as the carrier gas at a flow rate of 1.0 mL/min. The oven temperature program was as follows: initial temperature 40 °C held for 1 min, then raised to 150 °C at a rate of 4 °C/min and held for 6 min, then raised to 210 °C at a rate of 4 °C/min and held for 1 min. The ion source temperature was maintained at 280 °C, and mass spectra were acquired in electron ionization mode at 70 eV over the mass range of m/z 50–550. Compound identification was tentative and was performed by matching the obtained mass spectra and their fragments with the NIST mass spectral library (National Institute of Standards and Technology). Retention indices were calculated using a series of n-alkanes (C8–C30). A compound was considered positively identified when the NIST library match factor was ≥ 800.

#### Total phenolic content and antioxidant activity estimation

Folin-Ciocalteu method and was used to determine the total phenolic content as described by^34^ and the results were expressed as mg of gallic acid equivalent per mL oil (mg GAE/mL). Free radical scavenging capacities of essential oils were determined using the stable DPPH* and ABTS* according to^34^. Results were expressed as mg Trolox equivalents per mL oil (mg TE/mL).

#### Antimicrobial activity assay

Antimicrobial activities of cumin, cinnamon and clove essential oils were estimated against the tested bacterial and fungal strains using disk diffusion method according to^35,36^, respectively. The essential oils were prepared in DMSO at the concentration of 100 mg/mL and aliquot (20 *µ*L) of each sample was impregnated into the disk (6 mm diameter). The impregnated discs were placed onto solidified nutrient agar plates (NA) inoculated with 100 *µ*L of bacterial strains grown on Tryptic Soya Broth (0.5 Mc-Farland) or solidified potato dextrose agar plates (PDA) inoculated with 100 µL of fungal spore suspension. The NA plates were incubated at 37 °C for 18 h., while PDA plates were incubated at 28 °C for 24–48 h. DMSO was used as negative control. Ceftriaxone (1.0 mg/ mL) was used as positive antibacterial control and Miconazole (1.0 mg/mL) was used as positive antifungal. After incubation periods, the diameter of each inhibition zone was measured and expressed in mm. All experiments were performed in triplicate. It should be noted that inhibition zones of essential oils were not directly compared to those of standard antibiotics due to differences in diffusion properties and concentrations.

### Preparations and characterization of biopolymer-based films reinforced with CNFs

#### Film preparation

Fully hydrated CMC stock solution (3% w/v) was prepared by dissolving CMC in distilled water while stirring for 24 h to achieve a transparent solution with a moderate viscosity. In parallel, CNFs solution (2% w/v) was homogenized for 10,000 rpm for 3 min to avoid the agglomeration process. CMC stock solution was used as the control (CMC/0%CNFs), and different volumes of CMC and CNFs solutions were mixed to achieve a final solid concentration of 3%composed of CMC/2.5%CNFs, CMC/5%CNFs, CMC/10%CNFs and CMC/15%CNFs, following the separate addition and 30% glycerol were added based on total dry polymer solids (CMC + CNFs). Each film-forming solution was cast onto 50 mm diameter plastic Petri dishes, corresponding to a casting density of 0.2 mL/cm². The final total solids content of the films consisted of the aforementioned CMC, CNFs, and glycerol in ratios. The solutions were deaerated under vacuum and dried in desiccators containing saturated magnesium nitrate solution at 25 °C and ~ 54% RH for 72 h. The obtained films were peeled off, conditioned and the film thickness was measured at five random positions using a digital micrometer, and the average thickness was used for tensile calculations.

#### Film characterization

The mechanical properties including tensile strength, Young’ modulus and tensile strain of the prepared films were estimated using a Zwick-Z010 tensile testing machine at a crosshead speed of 12.5 mm/min according to ASTM D-882 standard methods. Before measurements, all films were conditioned at 23 °C and 50% RH for 24 h and then cut into 3 cm length and 1 cm width and analyzed in triplicate.

### Preparations of bioactive film forming solutions

Regarding the tensile properties and antimicrobial activity of essential oils, six CMC/10% CNFs film forming solutions were prepared. The first two solutions were prepared using CNFs from pomegranate (P-CNF) and sunflower heads (S-CNF) without essential oils. The third and fourth solutions were prepared by incorporating clove essential oil to P-CNFs (P-clove) and S-CNFs (S-clove) systems, respectively. The last two solutions were prepared by adding cinnamon and cumin essential oil mixture (1:1) into P-CNFs (P-cinnamon/cumin) and S-CNFs (S-cinnamon/cumin) systems, respectively. Essential oils (2% of polymer mass) were added dropwise to the CMC/CNF aqueous dispersions under continuous magnetic stirring. The aqueous CMC/CNF mixtures were further subjected to high-shear homogenization using at 10,000 rpm for 5 min to obtain stable emulsions.

### Manufacturing and evaluation of coated pan bread

#### Manufacture of bread

Pan bread was processed according to the straight dough method as described by^37^. The dough formulation consisted of wheat flour (72% extraction, 100 g), water (60 mL), sugar (4 g) active dry yeast (2 g), salt (2 g) and sunflower oil (2 g). All ingredients were mixed in lab kneader at low speed (30 rpm) for 4 min, followed by high speed mixing (60 rpm) for an additional 6 min. The bulk dough was then rested for 20 min at 30 °C, divided into 100 g pieces, molded, placed into metal pans (internal dimensions: 12 × 6 × 5 cm; length × width × height) and proved at 36 °C for 60 min. Bread loaves were baked in an electric oven at 240 °C for 20 min with steam applied during the first 7 min of baking.

#### Coating of bread

The bread loaves were separated from the metal pans, allowed to cool for 30 min and divided into seven groups, each of five loaves. The first group was set as the control group without any treatment. The edible film forming solutions (P-CNFs, S-CNFs, P-clove, S-clove, P-cinnamon/cumin and S-cinnamon/cumin) were individually applied on the loaves of each group by uniform brushing. Coating pickup was calculated for each loaf based on its volume. The average volumes ranged from 315.0 to 321.5cm^3^, corresponding to approximately 15.75–16.075mLper loaf. The control and treated bread samples were then rapidly dried for 10 min at 60 °C in an air oven to accelerate the film formation.

#### Determination of weight and freshness losses

Weight loss of bread samples was determined by repeatedly monitoring the same 3 loaves from each treatment at 24, 48, and 72 h of storage under controlled room conditions (25 ± 2 °C, relative humidity 60 ± 5%) in open-air conditions without packaging. Freshness of bread samples was determined at zero time and after 72 h using alkaline water retention capacity (AWRC) as described by^38^. Loss of freshness was calculated as follows:

Loss of freshness (%) = [(Freshness value at zero time - Freshness value at 72 h)/Freshness value atzero time)] X 100.

#### Sensory evaluation

Sensorial characteristics of bread samples including symmetry of shape (5), crust color (10), break & shred (10), crumb color (10), crumb texture (15), mouth feel (10), taste (20) and aroma (20) were evaluated after 2 hof baking as described by^39^.Samples were randomized in serving order by three-digit code and rated by twenty semi-trained panelists of Food Technology Department members using single-blind protocol. Panelists were selected based on the prior experience in sensory evaluation and the absence of any health conditions that could affect sensory perception such as allergies and intolerances.

#### Microbiological analysis

Total viable as well as mold and yeast counts of bread samples at zero time and after 72 h of storage were determined as recommended by FDA^40^ using pour plate technique. Under aseptic conditions, ten grams of each bread samples was transferred to sterile screw-cap jar containing 90 mL of sterile 0.1% peptone solution and shake vigorously manually about 50 times to achieve the initial homogenized suspension. Allow it to stand for 3–5 min, then resuspend by shaking five times through a 30 cm arc immediately before preparing serial dilutions. Serial dilutions of samples (10^− 2^ to 10^− 7^) were prepared in 0.1% peptone solution then platted on NA and PDA plates (1 mL per plate). All dilutions should be completed in the appropriate media within 15 min of blending the sample. The NA plates were incubated at 35 °C for 48 h and used for determination of total viable count, while PDA plates were incubated at 28 °C for 5 days and used for mold and yeast count. After incubation periods, the growing populations were counted and the results expressed as Colony Forming Unit per gram (CFU/g).

### Statistical analysis

The experiments of antioxidant activity, antimicrobial activity, microbiological analysis, and freshness loss were conducted with independent replicates (*n* = 3), while sensory evaluation was conducted with *n* = 20 panelists. Prior to analysis, ANOVA assumptions were verified via normality of residuals and homogeneity of variances tests. The obtained results were analyzed using one-way ANOVA followed by Duncan’s multiple range test to compare means at a significance level of *p* < 0.05 using Assistat Software (Version 7.7). Weight loss data (*n* = 3) were analyzed with a treatment-by-time analysis. Results are presented as mean ± standard deviation, and significance levels are indicated in tables and figures where applicable; for Fig. 5, error bars represent standard deviation (SD).

## Results and Discussion

### Characterization of cellulose nanofibers

#### Morphological properties

**Fig. 1:**
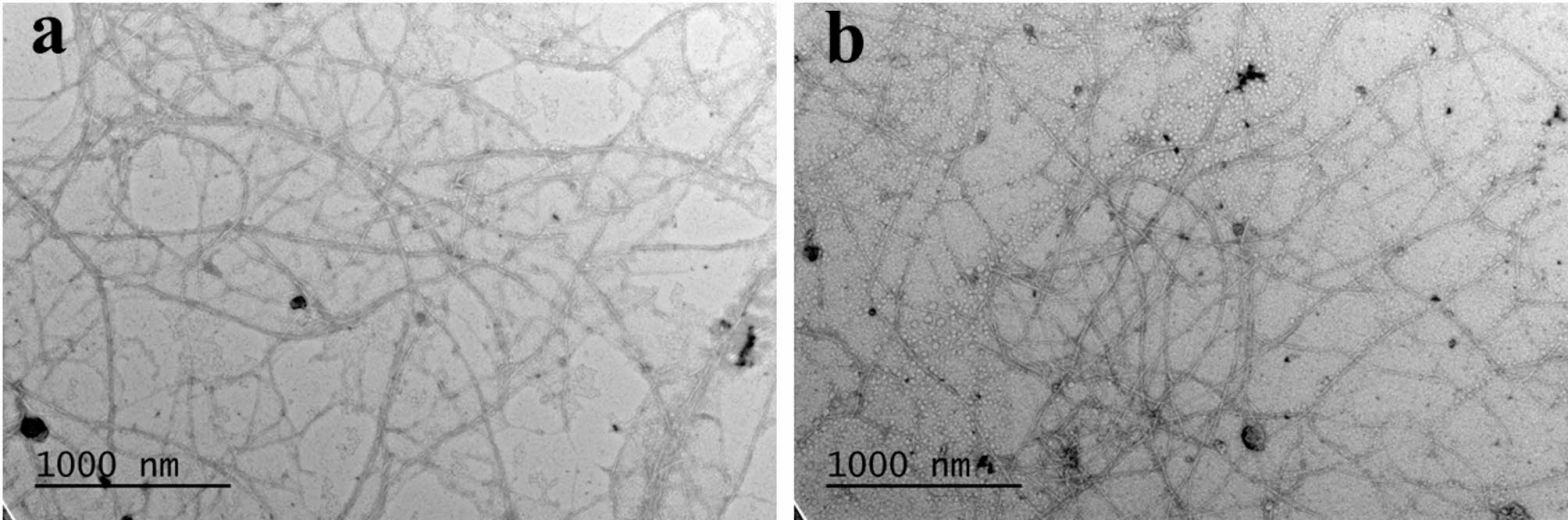
TEM of TCNF-SN (**A**) and TCNF-PP (**B**) prepared from sunflower and pomegranate peel respectively.

Transmission electron microscopy (TEM) was employed to investigate the morphology and dimensional characteristics of Tempo oxidized CNFs extracted from sunflower (TCNF-SN) heads and pomegranate peels (TCNF-PP). As illustrated in (Fig. 1), both CNFs samples exhibit a well-developed nanofibrillar structure with high uniformity and good dispersion. The isolated CNFs appeared as long, flexible, and entangled fibrils forming a continuous three-dimensional network, which is a typical morphological feature of well-fibrillated cellulose nanofibers. The TEM images clearly show that the CNFs obtained from both agricultural residues possess diameters in the range of 6–15 nm, while their lengths extend to several micrometers, resulting in very high aspect ratios^41^. Such nanoscale dimensions confirm the successful breakdown of the native lignocellulosic structure of sunflower heads and pomegranate peels into individualized cellulose nanofibrils.

The relatively narrow diameter distribution further indicates that the applied chemical pretreatment and fibrillation steps were efficient for both biomass sources. Moreover, the nanofibers were predominantly observed as individualized fibrils or small bundles, with no evidence of large aggregates. This morphological behavior highlights the effectiveness of the acidified sodium chlorite delignification process in removing lignin and hemicellulose fractions, thereby weakening the rigid plant cell wall matrix and facilitating fibril liberation^42^. In addition, the uniform and well-dispersed morphology of the CNFs can be attributed to the TEMPO-mediated oxidation process, which introduces negatively charged carboxylate groups onto the cellulose surface. These surface charges generate strong electrostatic repulsion forces between adjacent nanofibrils, effectively preventing re-aggregation during homogenization and TEM sample preparation. As a result, the CNFs maintain their individualized structure and form a stable nanofibrillar network^43^.

#### FT-IR analysis

**Fig. 2:**
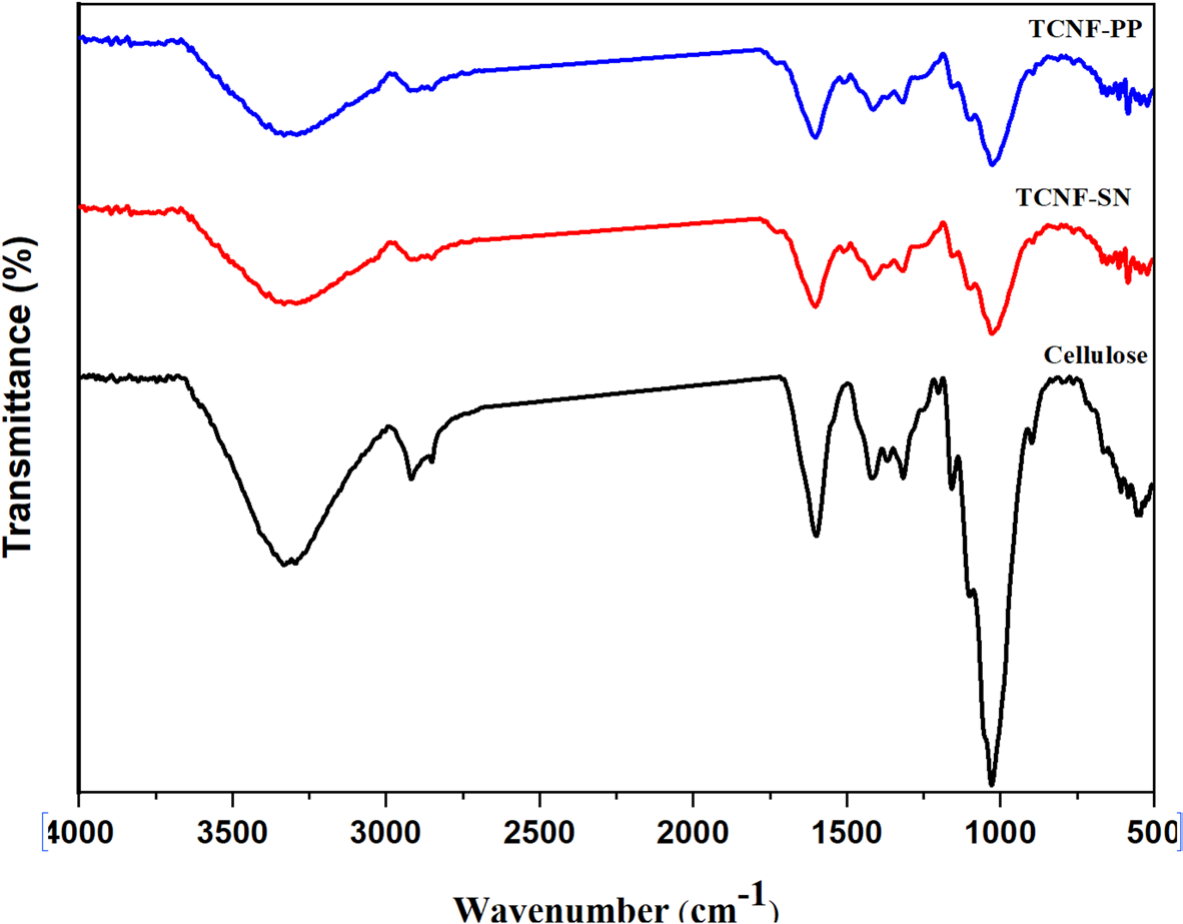
FTIR of cellulose, and TCNF- SN and TCNF-PPprepared from sunflower and pomegranate peel respectively.

FT-IR spectra of TCNF-SN and TCNF-PPas well as native cellulose are presented in (Fig. 2). The obtained spectra revealed characteristic peaks corresponding to cellulose bands, providing valuable insights into the chemical composition and functional groups present in the CNFs. Notably, several peaks of functional groups were observed in the spectra. The peak at approximately 3440 cm^− 1^ assigned to the stretching vibrations of the hydroxyl (-OH) groups in cellulose. Additionally, a band at 2928 cm^− 1^ was observed, which corresponding to the stretching vibrations of carbon-hydrogen (C-H) and methyl (-CH_2_) groups in cellulose. Another distinct peak appeared at 1015 cm^− 1^, could be attributed to the stretching vibrations of the C–O/C–O–C groups in cellulose. Furthermore, an interesting observation was in the spectrum of TEMPO-oxidized cellulose nanofibers (TCNF), where a new band emerged at approximately 1690 cm^− 1^ which can be associated with carbonyl stretching of carboxylic groups generated during oxidation. This band was interpreted as supportive evidence for TEMPO-mediated oxidation.

#### XRD analysis

**Fig. 3:**
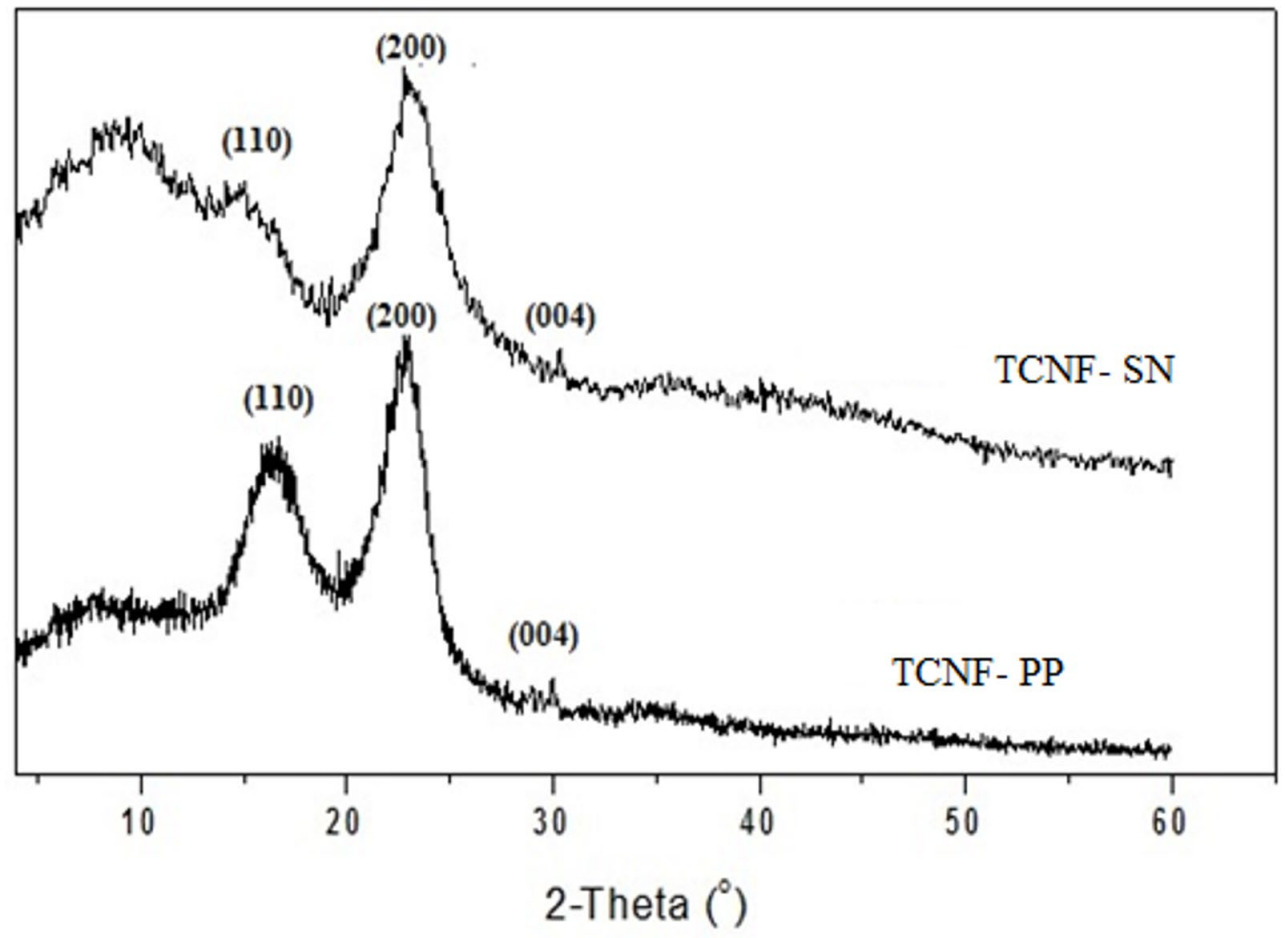
XRD diffractograms of TCNF- SN and TCNF-PPprepared from sunflower and pomegranate peel respectively.

The x-ray diffractograms of TCNF-SN and TCNF-PP, illustrated in (Fig. 3), revealed distinctive peaks corresponding to the characteristic crystallographic planes of native cellulose, namely (110), (200), and (004). These diffraction peaks provide insights into the crystalline structure of the cellulose nanofibers obtained from sunflower heads and pomegranate peels. In another words, the presence of these planes demonstrates the characteristic of cellulose I structure with no substantial changes in the position and shape of pristine cellulose peaks during oxidation process. This could be due to the oxidation resistance, low accessibility and high crystallinity of cellulose I crystals^44^.

To assess the degree of crystallinity, the Segal method was employed^45^. After TEMPO oxidation and nanofibrillation, the crystallinity indices of TCNF-SN and TCNF-PP increased to 69% and 72.5%, respectively, indicating a relatively high degree of crystallinity in both cellulose nanofiber samples. This increase in crystallinity may be attributed to the removal of amorphous components and less ordered regions during the extraction and preparation processes, including the partial elimination of hemicellulose and lignin^32^. The elimination of these amorphous regions leaves behind high crystalline cellulose with enhanced crystallinity percentage.

### Characterization of essential oils

#### GC-MS analysis

The volatile constituents of cinnamon, cumin, and clove essential oils were analyzed, and the dominant compounds are presented in Table 1. The essential oils studied showed different chemical constituents. Within the compounds listed in Table 1, clove essential oil was dominated by eugenol and eugenol acetate, whereas cinnamon and cumin essential oils were dominated by cinnamaldehyde and aldehydic/terpenoid constituents, respectively. Eugenol and cinnamaldehyde accounted for about 85.6% and 84.5% of clove and cinnamon oils, respectively. Similar results were reported for high concentrations of cinnamaldehyde and eugenol in cinnamon and clove oils^46,47^. In cumin essential oil, the main compounds 1,4-p-menthadien-7-al and cuminaldehyde accounted for 37.2% and 32.7%, respectively. Besides the distinct aroma, the presence of such bioactive constituents in essential oils is responsible for their antimicrobial and antioxidant properties^47^.

**Table 1:** Major compounds in cinnamon, cumin and clove essential oils.

Cinnamon		Cumin		Clove	
Compound	(%)	Compound	(%)	Compound	(%)
Cinnamaldehyde, (E)-	84.51	1,4-p-Menthadien-7-al	37.21	Eugenol	85.65
Cuminaldehyde	0.77	Cuminaldehyde	32.76	Caryophyllene	4.28
Copaene	4.2	1,3-p-Menthadien-7-al	8.61	α -Humulene	0.4
γ-Muurolene	0.63	γ -Terpinene	8.52	δ-Cadinene	0.04
α -Muurolene	2.24	(-)-β-Pinene	6.57	Eugenol acetate	9.53
δ-Cadinene	4.74	o-Cymene	5.26	Patchoulane	0.11
α -Copaene	0.79	1,3,8-p-Menthatriene	0.56		
Cubenol	0.65	3-p-Menthen-7-al	0.3		
τ -Muurolol	0.94	α -Pinene, (-)-	0.21		
δ-Cadinol	0.51				

#### Phenolic content and antioxidant activity

Total phenol contents and antioxidant activities of cinnamon, cumin and clove essential oils are presented in (Table 2). The analyzed samples showed different phenolic contents and antioxidant activities. Clove essential oil showed the highest phenolic content (73.3 mg GAE/mL) along with superior scavenging capacity against ABTS and DPPH radicals, recording 901.76 mg and 291.91 TE/ ml, respectively. Clove essential oil is well stated as a strong antioxidant agent due to its high phenolic content, particularly eugenol compound^48^. Although cumin essential oil showed lower content of total phenols (6.95 mg GAE/ml) compared to cinnamon oil (10.97 mg GAE/ ml), it showed higher antioxidant activity against both DPPH and ABTS radicals. This result could be attributed to the presence of cuminaldehydes and γ -terpinene, which act as potent hydrogen and electron donors, thereby contributing to its enhanced radical scavenging capacity^47^.

It was noteworthy that all tested oils displayed higher scavenging capacity against ABTS radicals compared to DPPH radicals. These results could be due to the fact that essential oils have a wide variety of bioactive ingredients, including terpenes and phenolic compounds. Consequently, essential oils could be more effective in the ABTS assay, which measures the scavenging capacity of both electron and hydrogen donating agents in aqueous or organic media. Whereas, the DPPH assay was conducted exclusively in organic media and primarily measured hydrogen-donating capacity^49,50^.

**Table 2:** total phenol contents and antioxidant activities of essential oils.

Sample	Total phenols (mg GAE/mL)	Antioxidant activity (mg TE/mL)	
		DPPH	ABTS
**Cinnamon**	10.97 ± 0.33	10.00 ± 0.30	19.14 ± 0.57
**Cumin**	6.95 ± 0.28	12.49 ± 0.50	52.71 ± 2.11
**Clove**	73.30 ± 1.17	291.91 ± 4.67	901.76 ± 14.43

#### Antimicrobial activity

The antimicrobial activity of cinnamon, cumin and clove essential oils against the tested bacterial and fungal strains was estimated and presented in **(**Table 3). Significant differences (*p* > 0.05) were observed between the antimicrobial activities of cinnamon, cumin and clove essential oils against the tested microorganisms. Except *S. aureus*, cinnamon essential oil showed the strongest antimicrobial activity against the tested microorganisms. Moreover, cinnamon oil exhibited the highest antimicrobial activity against all tested bacterial strains, except *S aureus*. *E. coli* was the most sensitive organism with inhibition zone diameter (IZD) 26.33 mm, while *C. albicans* and *A. flavus* were the most sensitive fungal strains toward cinnamon essential oil (IZD = 24.67 mm).

Clove essential oil showed strong antimicrobial activity against all tested strains. Clove oil outperformed cinnamon oil against *S. aureus*. *Bacillus cereus* was the most sensitive bacterial strains toward clove oil (IZD = 19.33 mm), while *A. flavus* was the most sensitive fungi with IZD of 21.33 mm. On the other hand, cumin essential oil showed the lowest antimicrobial activity compared to cinnamon and clove essential oils. *Salmonella typhi* was the most sensitive bacterial strains toward cumin oil (IZD = 15.67 mm) and *A. flavus* was the most sensitive fungal strain (IZD = 13.33 mm).

With respect to microbial strains, the results obtained are in a good agreement with the previous investigation of^51^ regarding the antimicrobial activity of cinnamon, clove, peppermint and black cumin essential oils. They stated that cinnamon and clove essential oils were the most effective antibacterial, with MBC values ranging from 0.0156 to 0.125 and from 0.25 to 1 ml/ml, respectively. Also, in the study of^52^ cinnamon oil was the most potent among 21 essential oils against 10 *Pseudomonas* species. Furthermore, in a comparative study between the effect of cinnamon and clove oils on the main causative agent of dental caries (oral microbiota)^22^, showed that cinnamon oil produced a maximum IZD of 24.0 mm against *Streptococcus mutans* compared to 13.0 mm for clove oil.

**Table 3:** Antimicrobial activity of cinnamon, cumin and clove essential oils.

Microorganism	Inhibition zone developed by essential oils (mm)				LSD
	Cinnamon	Cumin	Clove	*Positive Control	
**Bacteria**					
*Bacillus cereus*	24.67^A^	13.67^C^	19.33^B^	13.33^C^	1.438
*Staphylococcus aureus*	11.33^E^	10.33^C^	13.33^B^	16.67^A^	1.087
*Listeria monocytogenes*	17.33^A^	10.33^D^	13.00^C^	17.33^A^	0.941
*Salmonella typhi*	22.67^A^	15.67^B^	16.67^B^	12.67^C^	1.438
*Escherichia coli*	26.33^A^	13.33^C^	19.00^B^	25.00^A^	1.803
*Pseudomonas aeruginosa*	13.33^A^	8.67^C^	11.33^B^	13.33^A^	1.087
**Yeast and fungi**					
*Candida albicans*	24.67^A^	10.67^C^	15.67^B^	16.00^B^	0.941
*Aspergillus flavus*	24.67^A^	13.33^C^	21.33^B^	22.67^B^	1.718
*Aspergillus ochracious*	22.33^A^	10.67^D^	20.33^B^	16.67^B^	1.087
*Aspergillusniger*	21.00^A^	9.67^D^	19.33^B^	16.33^C^	1.331
*Fusariumproliferatum*	20.67^A^	11.33^D^	18.33^B^	16.33^C^	1.087
*Fusariumvertisoloides*	20.33^A^	11.00^D^	17.67^B^	16.00^C^	1.215
*Penicillium verrucosum*	21.33^A^	11.33^C^	19.00^B^	21.00^A^	1.215

*Ceftriaxone and Miconazole (1 mg/ml) were used as the positive antibacterial and antifungal control.

Means followed by different superscript letters in the same row are significantly (*p* > 0.05) different.

Several authors investigate the protective mechanisms of essential oils against a broad spectrum of pathogenic and spoilage microorganisms. The type and quantity of essential oils constituents are the main causative of their antimicrobial activity. In cinnamon oil, cinnamonaldehyde which is the major constituent could inhibit cell wall biosynthesis and disrupt membrane function and enzymatic activity of microbial cells^53^. Antimicrobial mechanisms of eugenol in clove oil include inhibition of microbial migration, adhesion, colonization, metabolism, invasiona nd suppressed the movement related gene expression^54^. Behbahani et al.^48^ stated that the phenolic compounds in cumin oil could inhibit enzymatic oxidation or modify protein functionality via non-specific interactions with sulfhydryl groups conferring its microbial growth-inhibiting mechanism.

However, the volatility of bioactive compound in some essential oils could limit the efficiency of these oils because it may be lost during estimation^55^. This could explain the lower antimicrobial activity of cumin essential oil and distinguish cinnamon and clove oils from the applied prospective.In general, the tested essential oils possess antimicrobial, antioxidant and hydrophobic properties, thereby could serve as natural bioactive ingredients in packaging materials.

### Mechanical properties of CMC/CNFs membranes

The mechanical properties of CMC films sunflowers derived CNFs at 0, 2.5, 5, 10 and 15% were investigated, and the results are shown in Table 4. The incorporation of CNFs into CMC films resulted in a pronounced enhancement in both tensile strength and Young’s modulus of the films. The tensile strength of the films increased progressively with CNFs loading up to 10%, with enhancements ranging from approximately 113.5% to 174%. The maximum tensile strength (28.5 MPa) was recorded at 10% CNFs, representing a 174% increase compared to the neat CMC film (10.4 MPa). Beyond this optimal loading, the tensile strength declined at 15% CNFs (27.4 MPa), indicating an over-reinforcement effect.

Similarly, Young’s modulus exhibited an upward trend with increasing CNFs concentration, reaching its maximum value of 1603 MPa at 10% CNFs, which corresponds to a 256% increase over the neat CMC film (450 MPa). At 15% CNFs loading, Young’s modulus slightly decreased to 1570 MPa, consistent with the decline observed in tensile strength. These findings are in good agreement with the previous investigation by Chaichi et al.^56^, who reported that the tensile strength of pectin-based films reinforced with crystalline nanocellulose (CNC) increased with CNC loading up to 5%, after which further reinforcement led to a decrease in mechanical performance.

The improved mechanical properties of CMC/CNFs films up to 10% loading suggest effective reinforcement of the CMC matrix. This enhancement may be attributed to favorable interactions, such as hydrogen bonding between the functional groups of CNFs and CMC molecules, as indicated by FT-IR analysis (Fig. 2). The close chemical structure of CMC and CNFs further promotes the formation of a continuous network of cellulosic whiskers via strong hydrogen and ionic interactions between the functional groups of CNFs and CMC molecules^57^. However, the decline in mechanical properties at 15% CNFs loading is attributed to nanofiber aggregation. At higher concentrations, the high surface area and strong inter-fibrillar hydrogen bonding promote the formation of CNFs agglomerates, leading to non-uniform dispersion within the CMC matrix. These aggregates act as stress concentration points, reducing the effective interfacial adhesion between CNFs and CMC and compromising the continuity of the reinforcing network. Similar observations have been reported for nanocellulose-reinforced pectin and starch films, where excessive filler loading leads to agglomeration and diminished mechanical performance^52,53^.

**Table 4:** Tensile properties of CMC films reinforced CNFs prepared from sunflowers.

Sample	Thickness	Tensile strength (MPa)	Young’s modulus (MPa)
**CMC/0% CNFs**	**0.2 mm**	10.4 ± 0.20	450 ± 02
**CMC/2.5% CNFs**	**0.2 mm**	22.2 ± 0.35	750 ± 25
**CMC/5% CNFs**	**0.2 mm**	25.7 ± 0.57	1450 ± 02
**CMC/10% CNFs**	**0.2 mm**	28.5 ± 0.50	1603 ± 03
**CMC/15% CNFs**	**0.2 mm**	27.4 ± 0.40	1570 ± 05

*CNFs prepared from sunflowers, values are presented as mean ± standard deviation (*n* = 3).

Several previous studies showed that there is an optimum reinforcement level of the filler to achieve the maximum tensile strength. The optimum level differs depending on the filler and matrix polymer type. For instance, the optimum reinforcing level of CNC in pectin film was 5%^56^, while the optimum level of CNFs in mango puree edible film was 10%^58^. Furthermore, García-Guzmán et al.^59^ studied the mechanical properties of starch films reinforced with CNC, CNFs and starch nanocrystals (SNCs) at 1 and 3%. They found that the nanoparticles of CNC and SNC at the level of 3% tend to agglomerate and hinder the reinforcement effect. However, this behavior was not observed in the case of CNFs, suggesting ease of CNFs dispersion than CNC and SNc particles.

### Effect of bioactive edible coating on the quality parameters of pan bread

#### Organoleptic properties

**Fig. 4:**
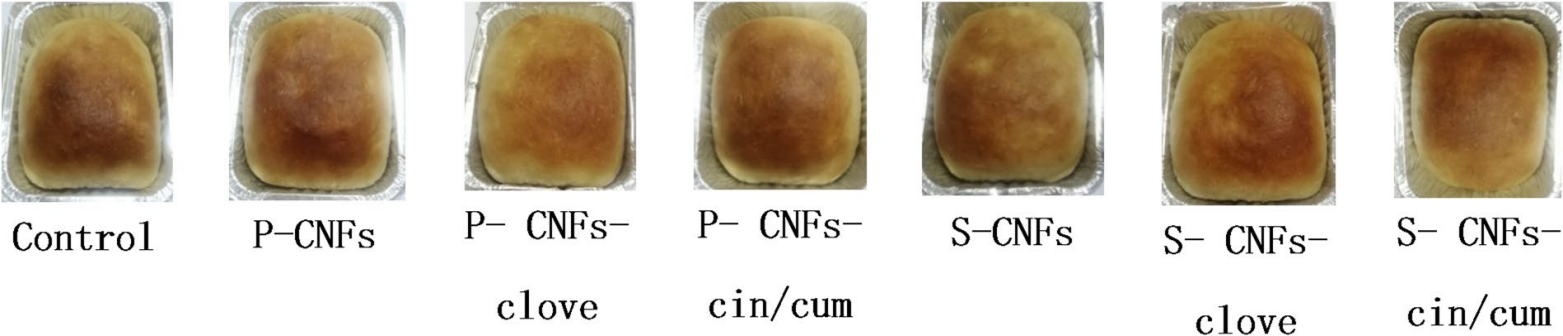
Photographs of bread samples coated with bioactive edible films. P-CNFs = pomegranate cellulose nanofibers, S-CNFs = sunflower head cellulose nanofibers, cin/cum = mixture of cinnamon and cumin oils.

Organoleptic properties of bread samples were evaluated by twenty semi-trained panelists that were asked to evaluate symmetry of shape, crust color, break & shred, crumb color, crumb texture, aroma, taste and mouth feel **(**Table 5**).** Data in this table showed that, except crust color, break & shred and aroma properties, were no significant differences (*p* > 0.05) between the organoleptic properties of bread samples. The crust color of coated bread samples gained higher scores compared to the control sample. The coated samples appeared shinier and more lustrous, especially those coated with the edible films containing essential oils (Fig. 4**).**

On contrary, beak & shred property of coated bread samples gained lower scores compared to the control bread because they were difficult to be sliced. This can be attributed to the formation of a surface barrier or film layer that enhances crust integrity and reduces crumb fragility. While such structural reinforcement may be beneficial in minimizing crumbling and mechanical damage during storage and transportation, it simultaneously leads to reduced sliceability, which is a critical quality attribute for consumer acceptance and industrial processing. From a technological standpoint, this trade-off is highly relevant. Bread that is difficult to slice may require greater mechanical force, potentially causing deformation of slices, uneven thickness, or tearing of the crumb. Therefore, future formulations should aim to balance structural reinforcement with sufficient crumb softness and elasticity to maintain acceptable break & shred characteristics, ensuring both enhanced shelf life and favorable processing performance.

**Table 5:** Organoleptic properties of breads coated with bioactive edible films.

Sample	Symmetry of Shape (5)	Crust color (10)	Break & shred (10)	Crumb color (10)	Crumb texture (15)	Aroma (20)	Taste (20)	Mouth feel (10)
**Control**	4.36	7.43^C^	8.57^A^	9.43	13.14	17.86^B^	16.86	9.00
***Bread coated with CMC/CNFs edible films***								
P-CNFs	4.43	7.71^BC^	7.43^B^	9.43	13.00	18.14^B^	17.14	9.14
S-CNFs	4.50	7.57^BC^	7.86^AB^	9.43	13.71	18.43^AB^	17.57	8.56
***Bread coated with CMC/CNFs edible films containing clove essential oil***								
P-CNFs	4.57	8.43^AB^	8.14^AB^	9.29	13.57	18.14^AB^	17.57	8.71
S-CNFs	4.43	8.43^AB^	8.00^AB^	9.86	13.86	19.00^A^	18.86	8.71
***Bread coated with CMC/CNFs edible films containing cinnamon and cumin essential oil***								
P-CNFs	4.36	8.43^AB^	7.86^AB^	9.29	13.43	17.86^AB^	17.00	8.57
S-CNFs	4.57	8.71^A^	8.00^AB^	9.43	13.43	19.00^A^	18.43	8.43
LSD	NS	0.962	0.780	NS	NS	1.093	NS	NS

Means followed by different superscript letters in the same column are significantly (*p* > 0.05) different.

P-CNFs = pomegranate cellulose nanofibers, S-CNFs = sunflower head cellulose nanofibers, LSD = least significant differences.

Regarding aromatic properties, the incorporation of essential oils produced varying effects. Bread samples coated with edible films containing either clove oil or a blend of cinnamon and cumin oils received significantly higher aroma scores and higher crust-color scores than the control. However, break and shred scores were lower or similar to the control, indicating that the coatings did not improve this attribute. The obtained results agree with those reported by Lotfinia et al.^60^ for bread samples coated with starch foams containing cinnamon oil. Furthermore, Aly et al.^61^ stated that coating muffins with edible films containing rosemary essential oil maintained the organoleptic properties of coated muffin samples up to 6 weeks. The edible coatings improved certain quality attributes such as appearance and aroma, careful formulation is required to minimize potential sensory drawbacks, particularly break and shred, to ensure full consumer acceptance and industrial feasibility.

#### Weight loss and staling properties of bread samples

**Fig. 5:**
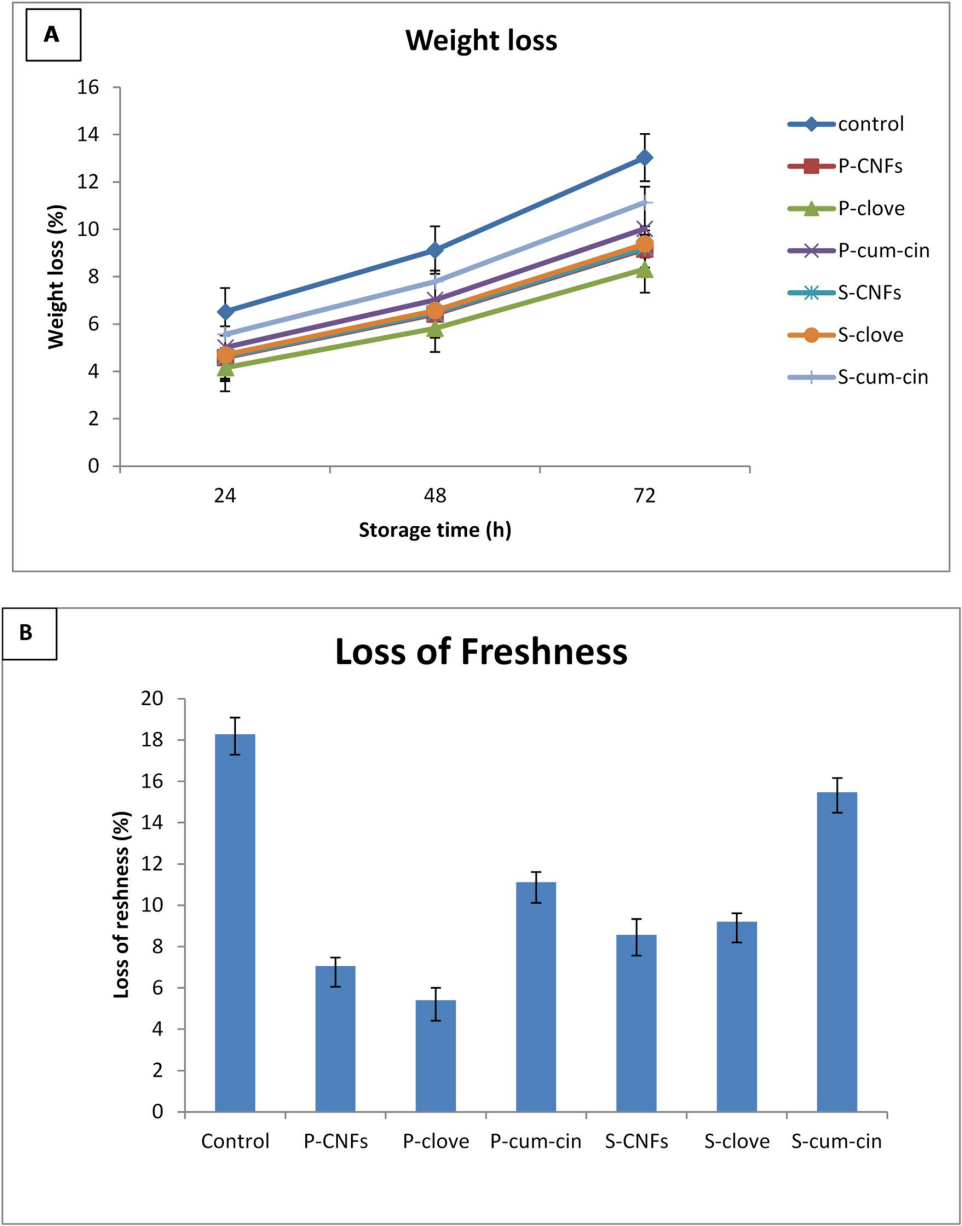
Weight and freshness losses of bread samples coated with film forming solutions during storage. Error bars represent standard deviation (SD, *n* = 3). P-CNFs = pomegranate cellulose nanofibers, S-CNFs = sunflower head cellulose nanofibers, cin/cum = mixture of cinnamon and cumin oils.

Due to the high initial moisture content of fresh bread (about 46%), weight loss was considered a practical indicator of moisture loss from bread during storage^62^. Therefore, the effect of coating solutions on weight loss and staling of bread samples during the storage period (3 days) was evaluated, and the results are illustrated in Fig. 5A. As expected, weight loss of all bread samples increased as the storage period increased, whereas weight loss of control bread was faster than that of the coated samples. The results obtained can be mainly attributed to moisture evaporation during storage of bread samples. Similar observations were reported by Yazdi et al. Yazdi et al.^30^, who showed that applying of chitosan-based coating formulations containing bacterial CNFs and silver nanoparticles on bread significantly improved their moisture retention during storage. Also, similar results were reported by Aly et al.^61^ for muffin coated with edible starch-based coatings containing rosemary essential oil.

Regarding the effect of film formulation on weight loss, bread samples coated with CMC film-forming solution containing pomegranate CNFs and clove oil showed the slowest weight loss rate. In contrast, bread samples coated with CMC solution containing cinnamon and cumin essential oils showed faster weight loss. These differences in weight loss may be tentatively attributed to possible variations in the water vapor barrier properties of the films, although water vapor permeability was not directly measured in this study. It is well established that lower water vapor permeability generally indicates greater resistance to moisture transmission, which can contribute to reduced weight loss^63^.

Previous studies have reported that hydrophobic compounds, including essential oils, can modify water transport in edible polymer films^64,65^. This behavior is commonly associated with the hydrophobic nature of essential oil components, which may alter the interaction between the polymer matrix and water molecules and contribute to reduced moisture migration through the coating layer^66^. Similarly, Bautista-Espinoza et al.^67^ developed edible coatings from quinoa protein and chitosan containing cinnamon and lemongrass essential oils encapsulated via mesoporous silica nanoparticles. They stated that the hydrophobic nature of the encapsulated oils reinforced the biopolymer matrix and decreased the trapped air bubbles resulting in less porous films. Furthermore, the drying process included in bread coating drives the oil droplets to be tightly packed in the polymer matrix^68^.

On the other hand, it is well known that high moisture release from the crumb to the surface of bread increases the staling process, resulting in decreased shelf life^69^. Application of edible films is accompanied by protection against drying deteriorations and of freshness loss reduction during storage^70^. The effects of film forming solution on bread staling were evaluated by AWRC and the results are illustrated in Fig. 5B. The trends of freshness loss were very similar to weight loss, where the control sample showed the highest staling rate. CMC film forming solutions containing pomegranate CNFs and clove oil showed the highest efficiency to maintain bread freshness during storage, while CMC solution containing cinnamon and cumin essential oils showed lower ability.

Considering the effect of moisture on bread freshness, maintaining the moisture content of bread could explain the ability of coating films to mitigate bread staling^30^. Likewise, Chen et al.^62^ and Mouzakitis et al.^71^ reported that the delayed crumb firmness of bread samples coated with hydroxypropyl methyl cellulose oleogel or zein films was due to prevention of moisture migration from crumb to crust. In general, data in Fig. 5 suggest that the incorporation of essential oils may have contributed to improved barrier properties of the polymeric coatings, which could help retard both weight and freshness loss of bread.

#### Microbial quality of bread samples

**Fig. 6:**
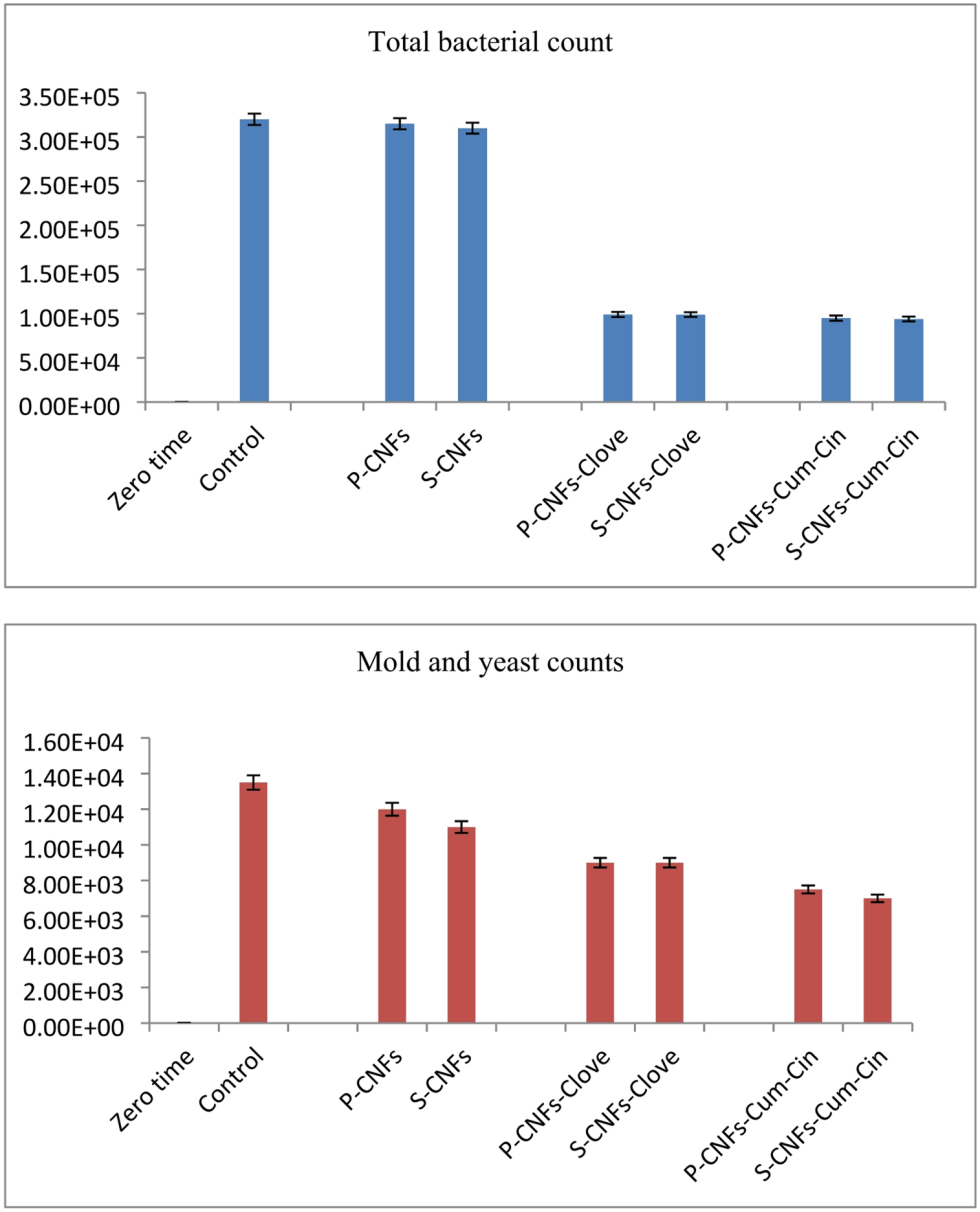
Microbiological properties of bread samples coated with film forming solutions. P-CNFs = pomegranate cellulose nanofibers, S-CNFs = sunflower head cellulose nanofibers, cin/cum = mixture of cinnamon and cumin oils.

The impact of coating solutions on the microbiological quality of bread samples at zero time and at the end of the storage period (72 h) at ambient temperature is illustrated in Fig. 6. The zero-time count (bacterial count 8.0 × 10 CFU/g and mold count 2.0 × 10 CFU/g) was low due to the high baking temperature. The bacterial count of bread samples ranged from 9.4 × 10^4^ to 3.2 × 10^5^ CFU/g at the end of storage. The control (uncoated) bread sample showed the highest count (3.2 × 10^5 CFU/g). Bread samples coated with CMC solutions reinforced with pomegranate-CNFs or sunflower-CNFs also recorded high bacterial counts (3.2 and 3.1 × 10^5^ CFU/g, respectively). In contrast, bread samples coated with CMC solutions containing pomegranate-CNFs or sunflower-CNFs combined with cinnamon/cumin essential-oil mixture showed the lowest bacterial counts (9.5 and 9.4 × 10^4^ CFU/g, respectively), which were lower than the uncoated control after 72 h. However, these values are close to 10^5^ CFU/g; therefore, the antimicrobial effect should be interpreted as a relative reduction at 72 h rather than complete microbial inhibition.

Similarly, the control bread sample showed the highest mold and yeast count (1.4 × 10^4^ CFU/g), followed by bread samples coated with CMC solutions containing pomegranate-CNFs or sunflower-CNFs (1.2 and 1.1 × 10^4^ CFU/g, respectively). The impact of essential oils was more pronounced on retarding fungal growth, as the coating solutions containing clove oil (9.0 × 10^3^ CFU/g). Likewise, CMC solutions containing pomegranate-CNFs or sunflower-CNFs accompanied with cinnamon and cumin essential oils showed the lowest mold and yeast counts (7.5 and 7.0 × 10^3^ CFU/g, respectively). According to Egyptian standards, the acceptable limits of total bacterial count and mold and yeast count of baked products, including bread, was set to not exceed 10^5^ and 10^4^ CFU/g, respectively^61^. Even though, the microbial counts of bread samples coated with CMC solutions reinforced with pomegranate-CNFs or sunflower-CNFs exceeded the limits of Egyptian standards indicating its weak antimicrobial activity. Instead the applied coating solutions incorporated with essential oils maintain the microbial populations within limits.

These results indicate that the antimicrobial activity of coating solution is mainly due to the effect of essential oils. In general, it was observed that the microbial count results of bread samples agree with the results of antimicrobial activity of the tested essential oils (Table 3). Among the tested oils, cinnamon essential oil showed the strongest antimicrobial activity, which could be due to its high content of cinnamaldehyde compound. The amount of this compound in essential oil used in the study was 84% (Table 1). Cinnamaldehyde is widely studied in several bioactive antimicrobial packaging films against a broad range of bacteria and fungi^72^.

Cinnamon oil showed the largest inhibition zones against most tested microorganisms (e.g., 26.33 mm against E. coli, 24.67 mm against Bacillus cereus, and more than 20.0 mm for the tested fungi), followed by clove oil and cumin oil with comparatively weaker activity. These in vitro results are consistent with the microbial counts recorded in bread samples, where formulations containing the cinnamon/cumin mixture showed lower total viable counts and mold/yeast counts than the uncoated control after 72 h. Thus, integration of essential oils, especially the cinnamon/cumin treatment delay microbial spoilage during the storage period. It is worth noting that inhibition zone diameters do not directly translate to equivalent antimicrobial performance in food matrices due to differences between agar and bread matrices, as well as interactions of essential oils with food constituents. The obtained results are in agreement with those reported by Bautista-Espinoza et al.^67^ and Wang et al.^73^ for cinnamon, bay, clove, thyme, and lemongrass essential oils, which were described as promising natural antifungal agents for food preservation.

## Conclusion

Pomegranate peels and sunflower heads represent sustainable sources for valuable intermediate substances such as CNFs, which were successfully extracted and functionalized through TEMPO-assisted oxidation methods at the nanoscale (6–15 nm in diameter). Antioxidant activity was measured for the essential oils, while antimicrobial activity was evaluated for both essential oils and coated bread samples. As a preliminary application, coated bread samples demonstrated that selected CMC/CNF/essential oil film forming solutions can contribute to maintaining product quality during short-term (72 h) open-air storage. In particular, the CMC film-forming solution containing pomegranate CNFs and clove oil was the most effective in retarding weight and freshness losses of coated bread samples, while the CMC film-forming solution containing the cinnamon/cumin oil mixture was the most effective in lowering microbial counts relative to the uncoated control after 72 h. Overall, this study highlights the potential of agro-waste-derived CNFs and essential-oil-incorporated CMC films as short-term bioactive food-coating materials, while emphasizing the need for further studies addressing EO-film mechanical properties, water-vapor barrier properties, essential-oil retention, migration/safety, and longer storage performance.

## Data Availability

All data generated or analyzed during this study are included in this published article.
